# Religious affiliation as a driver of immunization coverage: Analyses of zero-dose vaccine prevalence in 66 low- and middle-income countries

**DOI:** 10.3389/fpubh.2022.977512

**Published:** 2022-10-25

**Authors:** Thiago M. Santos, Bianca O. Cata-Preta, Andrea Wendt, Luisa Arroyave, Daniel R. Hogan, Tewodaj Mengistu, Aluisio J. D. Barros, Cesar G. Victora

**Affiliations:** ^1^Postgraduate Program in Epidemiology, International Center for Equity in Health, Federal University of Pelotas, Pelotas, Brazil; ^2^Programa de Pós-Graduação em Tecnologia em Saúde, Pontifícia Universidade Católica do Paraná, Curitiba, Brazil; ^3^Gavi, The Vaccine Alliance, Geneva, Switzerland

**Keywords:** vaccination, immunization, religion, inequality, global health, socioeconomic factors, diphtheria-tetanus-pertussis vaccine

## Abstract

**Background:**

The literature on the association between religion and immunization coverage is scant, mostly consisting of single-country studies. Analyses in low and middle-income countries (LMICs) to assess whether the proportions of zero-dose children vary according to religion remains necessary to better understand non-socioeconomic immunization barriers and to inform interventions that target zero-dose children.

**Methods:**

We included 66 LMICs with standardized national surveys carried out since 2010, with information on religion and vaccination. The proportion of children who failed to receive any doses of a diphtheria-pertussis-tetanus (DPT) containing vaccine – a proxy for no access to routine vaccination or “zero-dose” status – was the outcome. Differences among religious groups were assessed using a test for heterogeneity. Additional analyses were performed controlling for the fixed effect of country, household wealth, maternal education, and urban-rural residence to assess associations between religion and immunization.

**Findings:**

In 27 countries there was significant heterogeneity in no-DPT prevalence according to religion. Pooled analyses adjusted for wealth, maternal education, and area of residence showed that Muslim children had 76% higher no-DPT prevalence than Christian children. Children from the majority religion in each country tended to have lower no-DPT prevalence than the rest of the population except in Muslim-majority countries.

**Interpretation:**

Analyses of gaps in coverage according to religion are relevant to renewing efforts to reach groups that are being left behind, with an important role in the reduction of zero-dose children.

## Introduction

Monitoring health inequalities is at the core of the Sustainable Development Goals (SDGs). SDG 17.18 calls for national statistics to be “disaggregated by income, gender, age, race, ethnicity, migratory status, disability, geographic location and other characteristics relevant in national contexts” (1). Although the global literature on child health includes numerous analyses of inequalities according to wealth, gender, and geographic location, there are fewer studies on other dimensions of inequality. Religion fails to be mentioned in the SDG 17.18, even though belief systems can have direct implications on health seeking behavior, including vaccination.

Religious beliefs can negatively affect vaccination uptake through perceived theological objections to vaccination (2). Additionally, studies have found associations between religion and factors such as women's empowerment (3) which are known to be associated with vaccination coverage (4). The literature on religion and health has shown that maternal and child mortality is higher in Muslim-majority countries compared to non-Muslim-majority countries, and coverage of maternal and child health services is lower in Muslim-majority countries, with the discrepancy being attributed to contextual factors such as conflict, poor governance, and low female empowerment (5). Understanding these barriers can inform interventions meant to increase uptake of vaccination in zero-dose communities, thereby increasing vaccination coverage among zero-dose children.

Immunization is one of the most cost-effective interventions for reducing child mortality (6) and SDG indicator 3.b.1 reports on the proportion of the children covered by all vaccines in their national program. As part of the SDG focus on “leaving no one behind”, growing attention is being given to children who fail to receive any doses of routine vaccinations, the so-called “zero-dose children” (7). The Immunization Agenda 2030 includes a specific global target of reducing the number of zero-dose children by 50% by 2030 (8). To better identify such children, the community is focusing on no-DPT children, i.e., those without any doses of a diphtheria-pertussis-tetanus-containing vaccine, an indicator that may also be measured using data from administrative health information systems (7, 9–11). We will use the terms “zero-dose” and “no-DPT” interchangeably in this article.

The global literature on the association between religion and immunization coverage is scarce, mostly including reports from single countries such as Ethiopia (12) and Chad (13). In particular, we were unable to find any studies on zero-dose prevalence according to religious affiliation. We located a single multicountry analysis on religion and immunization coverage, covering 15 countries from sub-Saharan Africa suggesting lower coverage among Muslim children compared to other children, with the differences persisting even after accounting for sociodemographic factors (14). The study showed similar associations between religion and coverage for boys and girls.

This study builds on this literature to look at the association across 66 low and middle-income countries (LMICs) and explores the links between religion and zero-dose status. We also investigated whether these associations remained after adjusting for socioeconomic characteristics, and whether the associations persisted within different socioeconomic groups. Lastly, we explored whether children belonging to the majority religion in a country were more likely to be immunized than other children in the same country.

## Materials and methods

### Data sources

The International Center for Equity in Health at the Federal University of Pelotas (Brazil) has compiled a database of over 450 national surveys with information on reproductive, maternal, newborn and child health and nutrition (www.equidade.org). The surveys included in the analyses were all Demographic and Health Surveys (DHS) and Multiple Indicator Cluster Surveys (MICS) with publicly available datasets that were carried out from 2010 onwards in LMICs and that provided information on child vaccination status and household religion. If more than one survey were available for the same country, we selected the most recent one. Both families of surveys rely on nationally-representative, multi-stage sampling of households in LMICs to collect information about women aged 15 to 49, their households, and their children. The data collection methodologies and questionnaires for DHS and MICS are highly comparable (15, 16).

### Study samples and immunization indicator

A total of 66 country surveys – 32 DHS and 34 MICS – had information on both variables of interest. The outcome indicator was no-DPT prevalence, or the proportion of children aged 12–23 months who failed to receive any doses of DPT. In some countries, measles vaccine is given at 15 or 18 months, and we studied children aged 15–26 (Kazakhstan) and 18–29 (Egypt and Jamaica) months, respectively, in these specific countries. For all the other countries, the study sample was children aged 12–23 months. Even though measles vaccine is not included in the no-DPT indicator, this was done to ensure consistency with global and national vaccine coverage reports. Information on immunization status came from two sources: vaccination cards or, when the child did not have a card or it was unavailable, the mother's or caregiver's report. We treated children with missing information on immunization as not vaccinated.

### Religious groups

Each survey collected data on religious groups that were common in the country. Information on religion in MICS is from the head of the household (household questionnaire) while in DHS is from women (woman's questionnaire). In our analyses, families were classified into seven groups: Muslim, Christian, Hindu, Buddhist, folk (or traditional), others and unaffiliated (14). Supplementary Table 5 presents the original religious groups and the categorization used in this study. Within each country, the “largest group” was defined as the one with the highest proportion in the sample. The “majority group” was defined as a religion that is practiced by 50% or more of the sample.

### Statistical analyses

First, we compared no-DPT prevalence between religions within countries. We opted to use Christians – the most frequent group overall – as the reference in the comparative analyses. We performed adjusted Wald tests between each pair of religions and restricted the analysis to those with at least 25 children.

Secondly, covariate-adjusted analyses were used to assess whether family wealth, maternal education or urban-rural residence explained associations between religion and immunization. We standardized all indicators in the DHS and MICS datasets and then merged data for all countries in order to perform the analyses. Households were classified into wealth quintiles based on an asset index derived through principal components analyses. The asset indices, which are provided in the DHS and MICS datasets, are calculated separately for urban and rural households on the basis of relevant assets and building characteristics in each context, and later merged into a single national index (17). Maternal education was classified into three groups (none, primary, and secondary or more) and place of residence was categorized as either urban or rural on the basis of national definitions. Countries were grouped into world regions using the UNICEF classification (18).

A Poisson regression with robust variance (19) was used in the analyses. The units of analysis were all children from all countries with data. Adjustments included fixed effects for countries and individual level covariates. In the pooled analyses, national results were weighted by the populations of children aged 12–23 months in 2016, (20) which was the median year of all surveys. Detailed information about sample weight adjustment for population of children in each country is presented in the Supplementary materials.

Lastly, we compared the no-DPT prevalence in children from the majority religions within a country and the rest of the population and investigated the intersectionality between socioeconomic position and religion in terms of no-DPT prevalence.

The analyses were carried out with R version 4.1.0 and Stata version 17 and accounted for the multi-stage survey design and sample weights.

## Results

**Figure 1:**
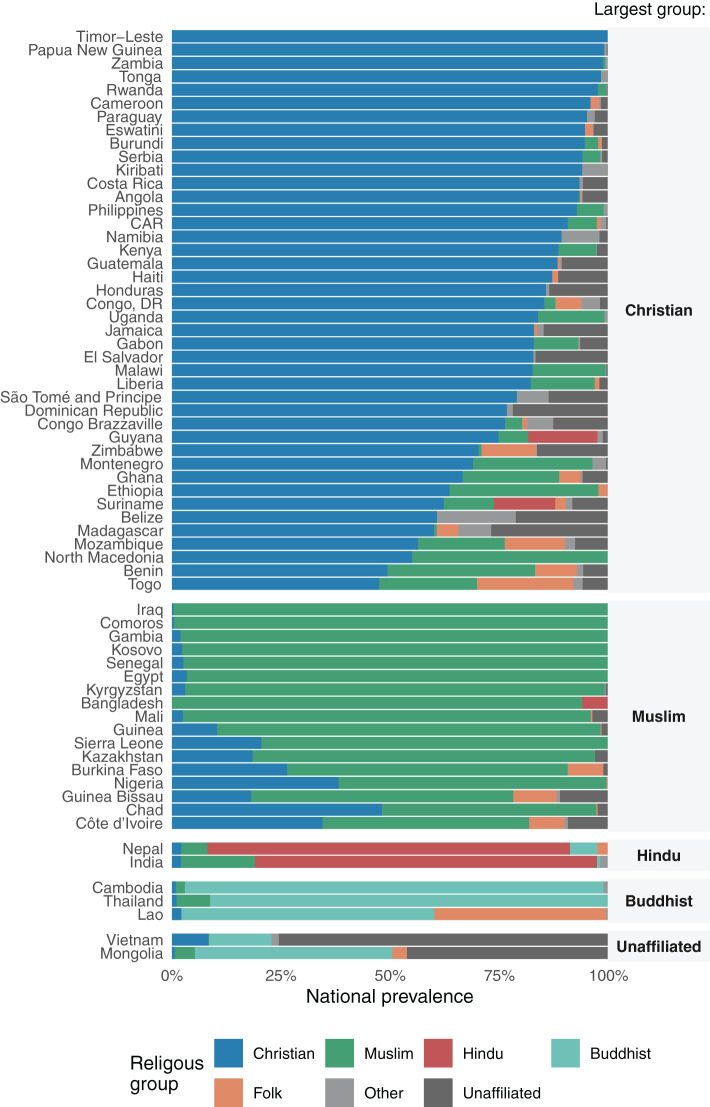
Distributions of the samples of children according to religious group, by country.

A total of 159,063 children from 66 countries were included in the analyses. The distribution of countries according to UNICEF regions was as follows: West and Central Africa (20), Eastern and Southern Africa (14), Middle East and North Africa (2), Eastern Europe and Central Asia (6), South Asia (3), East Asia and the Pacific (10) and Latin America and Caribbean (11). Figure 1 and Supplementary Table 1 show the distribution of religions in the national samples, stratified by the largest religious group in each country.

In 61 of the 66 countries, one religious group comprised over 50% of the children, as follows: Christian (40 countries); Muslim (15), Buddhist (3), Hindu (2), and unaffiliated (1). The remaining five countries did not have a majority religion. Folk religions were either small minorities or totally absent in the samples.

Supplementary Table 2 shows the average national no-DPT prevalence and 95% confidence intervals for each country and Supplementary Table 3 shows the no-DPT prevalence and sample size for each religious group by country.

When comparing no-DPT prevalence between religions within individual countries, we found significant heterogeneity in 27 of the 55 countries with at least two religious groups with 25 or more children. In 11 countries, no-DPT prevalence was significantly higher in Muslim children compared to Christian children, and in 3 countries it was lower. Children from folk religions had significantly higher and significantly lower no-DPT prevalence compared to Christians in 3 countries and 1 country, respectively. Similarly, unaffiliated children had significantly higher and lower no-DPT prevalence in five and three countries, respectively. Buddhists and other religions had significantly lower no-DPT prevalence than Christians in 1 country and no countries with higher no-DPT prevalence. Finally, there were no countries with significant differences between Hindu and Christian children in any country. Additional information is provided in Supplementary Table 4, which shows the number of countries with significant differences for each pair of religions.

In Table 1, the first column with data shows the unadjusted results based on analyses, with all 159,063 children from all countries. The lowest frequency of no-DPT children was observed among Buddhists, followed by Hindus, both of which had significantly lower prevalence of no-DPT compared to Christians. On the other extreme, folk religions and Muslims showed the highest frequency of no-DPT in the unadjusted analyses. These analyses are likely biased by national no-DPT prevalence levels in the few countries where some religions are common, as is the case for Hindus and Buddhists. The bias is removed when fixed effects for country are included in the model, as is shown in the second column. The reduced prevalence among Buddhists and Hindus was no longer present, with higher no-DPT prevalence in both cases relative to Christians and a significant difference for Hindus. Higher prevalence relative to Christians was also observed for Muslim and folk religions, as well as for the unaffiliated in the model with fixed effects.

**Table 1:** Poisson regression analyses with children as the units of analyses, including fixed effects for countries and covariates.

**Religious group**	**No-DPT prevalence ratio (95%CI)**			**Number of countries with data**
	**Crude**	**Adjusted using fixed effects for country**	**Adjusted using individual variables and fixed effects for country**	
Christian	1	1	1	
Muslim	**1·37 (1·27, 1·48)**	**2·37 (2·14, 2·63)**	**1·76 (1·59, 1·95)**	49
Hindu	**0·66 (0·62, 0·72)**	**1·34 (1·18, 1·53)**	1·05 (0·93, 1·19)	5
Buddhist	**0·39 (0·31, 0·50)**	1·27 (0·84, 1·91)	1·21 (0·81, 1·82)	8
Folk	**1·86 (1·58, 2·18)**	**1·81 (1·53, 2·14)**	**1·39 (1·20, 1·60)**	32
Other	0·99 (0·79, 1·24)	1·12 (0·91, 1·38)	1·04 (0·86, 1·26)	48
Unaffiliated	0·94 (0·83, 1·07)	**1·86 (1·67, 2·08)**	**1·44 (1·30, 1·60)**	52

Bold results signal 95% confidence intervals that do not include the unity.

The third column in Table 1 explores whether adjustment for household wealth, maternal education and area of residence explained the associations between religion and no-DPT. All prevalence ratios relative to Christians were attenuated by the adjustment, but children from Muslim, Unaffiliated and Folk religious groups remained associated with 76, 44, and 39% higher no-DPT prevalence respectively compared to Christian children.

The pooled analyses are informative but need to be supplemented by country-by-country analyses as the behaviors of religious groups are likely affected by national contexts (see Supplementary Table 2 for national results). In fact, Muslims in Congo Brazzaville, Mozambique, and Suriname, folk practitioners in Zimbabwe and the unaffiliated groups in Burkina Faso, Kazakhstan, and Zimbabwe had significantly lower no-DPT prevalence than Christians.

**Figure 2:**
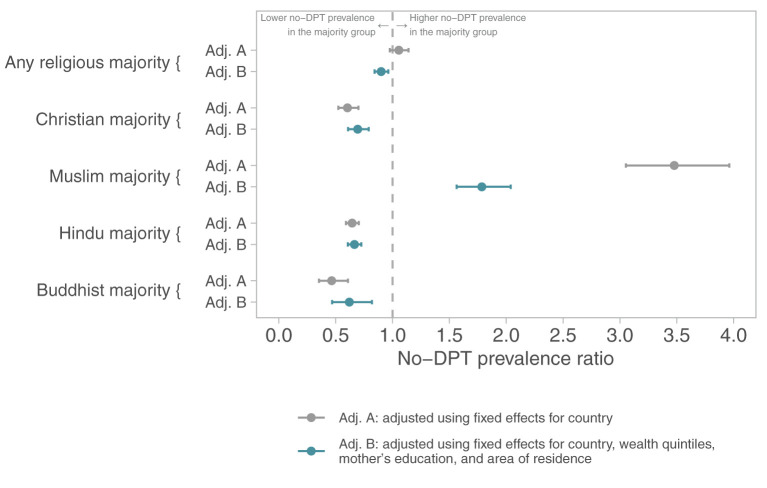
No-DPT prevalence ratios comparing the majority religion with remaining children in each country. Poisson regression models with children as the units of analysis, including fixed effects for country (upper lines) and adjusting for wealth, maternal education, and residence (bottom lines).

The next set of analyses (Figure 2) investigates whether majority religions within a country tend to have lower rates of zero-dose children than the rest of the population. Two lines are shown for each comparison: the first with fixed effects for country and the second with additional adjustment for household wealth, education, and area of residence. Majority religions in each country tended to have lower no-DPT prevalence than the rest of the population. The only exception were Muslim-majority countries where children from other religious groups tend to have lower no-DPT prevalence than Muslim children.

**Figure 3:**
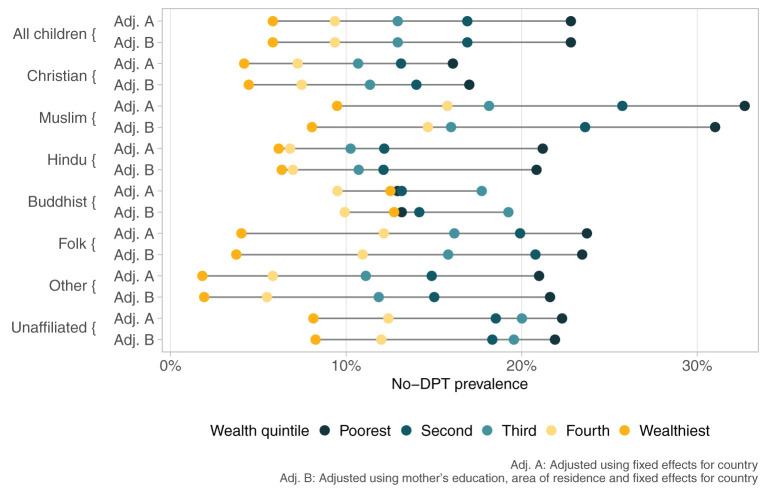
No-DPT prevalence according to religious group and wealth quintiles. Poisson regressions with children as the units of analyses, including fixed effects for country.

Lastly, Figure 3 explores the intersectionality between socioeconomic position and religion in terms of no-DPT prevalence, using data from all countries with information on each religion. Within any religious group, there are wide socioeconomic gaps, but their magnitude varies by religion, being most marked among Muslim children. Figure 3 also allows comparison of coverage within each wealth quintile for the different religions. In the poorest quintile, no-DPT prevalence ranged from 16·4% (95%CI 15·1;17·6%) in Christians to 33·4% (95%CI 31·6;35·2%) in Muslims, a gap of 17·0 percent points. Prevalence in the wealthiest quintile ranged from 4·5% (95%CI 4·0;5·1%) in Christians to 9·3% (95%CI 8·1;10·5%) in Muslims, a gap of 4·8 percent points. These findings show that higher socioeconomic position attenuated the gaps among religions.

## Discussion

The present study is the first to look at the relationship between religion and zero-dose status across a wide range of countries, providing a comprehensive examination of religious inequalities in child immunization. Our results are consistent with the existing literature, suggesting that religious affiliation can influence immunization decisions in certain contexts. We have found that children from Muslim households – as well as from Folk and religiously unaffiliated households, albeit to a lesser extent – had higher no-DPT prevalence compared to children from Christian households, even after controlling for national prevalence and household level factors. Even in Muslim majority countries, Muslim children were less likely to be vaccinated than children from religious minorities in the same country. In addition, socioeconomic gaps in vaccination coverage were wider for Muslim children compared to children from other religions. The only multi-country study on this topic, including 15 sub-Saharan African countries, had already reported that Muslim religion was associated with lower vaccine coverage in most countries studied, both in unadjusted analyses and after adjustment for wealth, education, and residence (14). Several single country studies also provide similar results.

To explain the gaps in immunization according to religion, the literature has pointed to active messages from religious leaders against vaccination, reliance on traditional healers, and/or resistance to Western medicine. In Africa – which includes most of the countries with high zero-dose prevalence – religious affiliation represents a major source of identity and social connection, with religious leaders having direct influence over the health decisions of members of their respective religious groups (11, 21).

In Nigeria, a country with one of the highest zero-dose prevalence, religious leaders play a significant role in individual attitudes, behaviors, and social norms which have historically influenced child health care, with strong assertions and speculations against vaccination. Nigeria has a complex healthcare system that mixes Western medicine with traditional healing practices such as herbalists, healers, and Islamic diviners. A child immunization campaign in 2003 had to be suspended in the northern states of the country due to a counter campaign in which Muslim leaders urged parents not to allow their children to be immunized. Claims were made that polio vaccines were contaminated with anti-fertility agents, carcinogens, and HIV, being part of a Western plot to reduce the Muslim population. An earlier study reported that lack of immunization was about 60% higher among Muslim than Christian children, even controlling for socioeconomic and women's empowerment indicators (21).

Our study also found higher zero-dose prevalence among children in folk religious groups compared to Christians. A mixed-methods study carried out in Kenya showed that practitioners of traditional African religions may be hesitant toward vaccination because of their assumption that a child is born immune, and that Western medicine interventions such as vaccination lead to pain, thus compromising the child's immune system (22). In addition, traditional healers offer the first line of health care for many families (22). Lower vaccine coverage for groups practicing traditional religions were also found in Burkina Faso, (23) where researchers highlighted that low coverage is not restricted to vaccines, but also applies to antenatal and institutional delivery care (24, 25). Affiliation with folk religions tends to be strongly related to ethnicity and socioeconomic characteristics. For example, in India, tribal populations often rely on traditional healers and there is resistance toward adopting Western biomedical interventions, which leads to vaccine hesitancy (26). Another study from Ghana showed that children whose mothers were traditionalists or unaffiliated presented approximately 40% lower odds of being fully immunized, compared to children whose mothers were Christians (27). This highlight the relevance of within-country analyses of religious gaps in coverage to identify groups being left behind.

The intersectionality analyses showed that socioeconomic gaps in coverage were wider for Muslim children than for other religions. They also showed that religion-related gaps were narrowest for children in the wealthiest quintile, and widest for those in the poorest quintile. A similar pattern has been reported in global comparisons of immunization coverage by wealth quintile, where variability among countries was markedly smaller for children from wealthier families than for children from poor families (28). This is an example of a “safety net” effect, as wealthier families are more likely to access health care irrespective of the country where they live (29).

There are important nuances to keep in mind when interpreting these results. First, vaccine hesitancy is not driven by theological basis, but rather beliefs within specific communities. A review of religious teaching that sought the scriptural, canonical basis for vaccine hesitancy in major religious groups found that reasons for declining immunization are generally not theologically based, but rather the result of beliefs among a network of people or communities organized around a faith (2). As a result, religious beliefs with respect to vaccination are complex and diverse within one religion. For example, a descriptive study conducted in Malaysia to measure the knowledge, attitude, and perception of parents toward vaccination found that the majority of Muslim parents strongly agreed with the need for vaccinating infants, and believed that “vaccines are not prohibited in Islam”; most Muslim parents also rejected the belief that “all vaccines are non halal and hence should be avoided” (30). The pooled analyses of a survey on vaccine confidence in 66 countries showed that Muslim respondents were about 30–40% less likely than Christians to answer that vaccines are important, safe, and effective. However, there was substantial variability among Muslims regarding whether they agreed with the statement that “vaccines are compatible with my religious beliefs”. In Saudi Arabia, all respondents were Muslim and 98% agreed with the statement, meanwhile Muslims in Nigeria and Pakistan showed moderate levels of objection; both countries having a history of rejection against polio vaccine linked to religious fundamentalism (31).

Second, the overall pattern described in the study is not present in every country. Religious communities are diverse in terms of beliefs, religious expressions, and engagement even in the community level, let alone from a global perspective. In our analysis, there were statistically significant difference in no-DPT prevalence between religious groups in only 27 of 55 countries. Also, as an example, in Congo Brazzaville, Mozambique, and Suriname, children from Muslim households had significantly lower no-DPT prevalence then children from Christian households.

Our analyses have limitations. First, our sample does not include high income countries and includes only 16 upper-middle income countries, and vaccine hesitancy is on the increase in several countries in these two categories (32). Second, in some countries the number of sampled children in specific religious groups was small, and these groups had to be removed from the analyses. Third, information regarding religion in DHS is obtained from the mother, while in MICS the question applies to the head of household; it is not possible to assess how this would affect the present results. Fourth, the covariates in the adjusted analyses – wealth and education – may be influenced by religious affiliation, and therefore represent mediating variables rather than confounders; if so, the adjusted results would underestimate the full effect of religion. Fifth, the broad religious groups used in the analyses may include subgroups with different behaviors and beliefs. For example, Christians may include Catholics, traditional protestants, and evangelicals, while Muslims may include Sunnis, Shias, and other sects. A detailed comparison of such subgroups is outside the scope of the present analyses, and in addition the survey questions often fail to provide such information. One should also note that our analyses include surveys carried out since 2010 (median year 2016) so that for some countries there may have been changes in coverage in the recent past. A final limitation is that the survey questionnaires do not allow us to investigate whether low coverage was due to health system shortcomings or due to choices made by the families.

Among the strengths of our analyses, these represent the most comprehensive examination of religious inequalities for a child health outcome. Including analyses of religion within the framework of social determinants of child immunization coverage complements more traditional inequality analyses focused on household wealth, parental education, or place of residence (33, 34).

In conclusion, our multi-country analyses expand upon previous publications on religious gaps in immunization in 15 African countries (14) and single-country reports, (21, 27, 35–38) all of which revealed important gaps according to religion in some countries, and specifically the finding that Muslim children are being left behind in many countries. Identification of religious gaps is likely essential for identifying delivery channels for health education regarding immunization in many countries. It also provides an important step for further in-depth investigations to elucidate the reasons for low coverage. Experience from Sierra Leone, Angola, and India suggests that involvement of Islamic leaders had positive effects on child immunization coverage, (39) indicating an important role for religious leaders and faith-based organizations in reaching zero-dose children.

At global level, immunization coverage increased until the 2000s, followed by a period of stagnation until the COVID-19 pandemic, when coverage dropped. For example, DPT3 coverage dropped from 86% in 2019 to 81% in 2021 (40). Efforts are urgently needed to counteract these recent declines. Within-country analyses of religious gaps in coverage and renewing efforts to reach groups that are being left behind could play an important role for reaching the Immunization Agenda 2030 global target of reducing the number of zero-dose children by 50% by 2030 (8).

## Data availability statement

All the analyses were carried out using publicly available datasets that can be obtained directly from the DHS (https://dhsprogram.com) the MICS (mics.unicef.org) websites.

## Ethics statement

Our analyses employed publicly available, anonymized databases from national surveys. Informed consent was obtained by the institutions that administered the surveys in accordance with local guidelines. Ethical approval for data collection was obtained in each country.

## Author contributions

TS, BC-P, AW, and LA conducted the analyses and verified the underlying data, with support from CV and AB. TS, LA, AW, and CV prepared the first draft of the manuscript, which was revised and edited by all other authors. All authors conceptualized the article, interpreted the results, read, and approved the final manuscript.

## Funding

This paper was made possible with funds from Bill and Melinda Gates Foundation (Grant Number: OPP1199234), Gavi, the Vaccine Alliance, Wellcome Trust (Grant Number: 101815/Z/13/Z), and Associação Brasileira de Saúde Coletiva.

## Conflict of interest

TM and DH are employed by Gavi, the Vaccine Alliance, sponsor of this research. They had total freedom to express their views which do not necessarily reflect those of Gavi, the Vaccine Alliance. The remaining authors declare that the research was conducted in the absence of any commercial or financial relationships that could be construed as a potential conflict of interest.

## Publisher's note

All claims expressed in this article are solely those of the authors and do not necessarily represent those of their affiliated organizations, or those of the publisher, the editors and the reviewers. Any product that may be evaluated in this article, or claim that may be made by its manufacturer, is not guaranteed or endorsed by the publisher.
