# Supplementary material for: Religious affiliation as a driver of immunization coverage: Analyses of zero-dose vaccine prevalence in 66 low- and middle-income countries
Source: Front Public Health. 2022 Oct 25;10:977512. doi: 10.3389/fpubh.2022.977512 (PMC9642099; doi:10.3389/fpubh.2022.977512)
Supplement: Supplementary file 1 [file Data_Sheet_1.docx]

Supplementary materials

# Sample weight adjustments for population 12-23 months

In order to take into consideration the number of children aged 12-23 months living in each country, individual sample weights were adjusted using the following equation:

$$w_{ij, adj}=\left( \frac{w_{ij}}{\sum_{i} w_{ij}} \right)\left( \frac{{pop}_{j}}{\sum_{j} {pop}_{j}} \right)N$$

where:

- *i* indicates a child and *j* a country
- $w_{ij, adj}$ is the adjusted individual sample weight
- $w_{ij}$ is the original sample weight
- ${pop}_{j}$ is the population of children aged 12-23 months in the country *j*
- *N* is the total number of children in the sample

Supplementary Table 1. Distribution of religious group in the national samples

| Country | Religious group | Proportion | 95% Confidence interval | | N |
| --- | --- | --- | --- | --- | --- |
|  |  |  | lower limit | upper limit |  |
| Angola | Buddhist | 0.0 | 0.0 | 0.0 | 2845 |
| Angola | Christian | 93.2 | 91.2 | 94.8 | 2845 |
| Angola | Folk | 0.3 | 0.1 | 0.7 | 2845 |
| Angola | Hindu | 0.0 | 0.0 | 0.0 | 2845 |
| Angola | Muslim | 0.3 | 0.1 | 0.6 | 2845 |
| Angola | Other | 0.3 | 0.1 | 0.5 | 2845 |
| Angola | Unaffiliated | 6.0 | 4.5 | 8.0 | 2845 |
| Bangladesh | Buddhist | 0.2 | 0.1 | 0.6 | 1666 |
| Bangladesh | Christian | 0.0 | 0.0 | 0.3 | 1666 |
| Bangladesh | Folk | 0.0 | 0.0 | 0.0 | 1666 |
| Bangladesh | Hindu | 5.9 | 4.5 | 7.6 | 1666 |
| Bangladesh | Muslim | 93.9 | 92.1 | 95.3 | 1666 |
| Bangladesh | Other | 0.0 | 0.0 | 0.0 | 1666 |
| Bangladesh | Unaffiliated | 0.0 | 0.0 | 0.0 | 1666 |
| Belize | Buddhist | 0.0 | 0.0 | 0.0 | 502 |
| Belize | Christian | 60.7 | 54.9 | 66.2 | 502 |
| Belize | Folk | 0.0 | 0.0 | 0.0 | 502 |
| Belize | Hindu | 0.0 | 0.0 | 0.0 | 502 |
| Belize | Muslim | 0.0 | 0.0 | 0.0 | 502 |
| Belize | Other | 18.0 | 14.0 | 23.0 | 502 |
| Belize | Unaffiliated | 21.3 | 16.6 | 26.8 | 502 |
| Benin | Buddhist | 0.0 | 0.0 | 0.0 | 2522 |
| Benin | Christian | 49.4 | 46.3 | 52.5 | 2522 |
| Benin | Folk | 9.5 | 7.8 | 11.6 | 2522 |
| Benin | Hindu | 0.0 | 0.0 | 0.0 | 2522 |
| Benin | Muslim | 33.8 | 30.9 | 36.8 | 2522 |
| Benin | Other | 1.5 | 0.9 | 2.3 | 2522 |
| Benin | Unaffiliated | 5.8 | 4.8 | 7.2 | 2522 |
| Burkina Faso | Buddhist | 0.0 | 0.0 | 0.0 | 2777 |
| Burkina Faso | Christian | 26.4 | 23.8 | 29.2 | 2777 |
| Burkina Faso | Folk | 8.1 | 6.4 | 10.2 | 2777 |
| Burkina Faso | Hindu | 0.0 | 0.0 | 0.0 | 2777 |
| Burkina Faso | Muslim | 64.2 | 61.1 | 67.3 | 2777 |
| Burkina Faso | Other | 0.0 | 0.0 | 0.2 | 2777 |
| Burkina Faso | Unaffiliated | 1.2 | 0.7 | 2.2 | 2777 |
| Burundi | Buddhist | 0.0 | 0.0 | 0.0 | 2596 |
| Burundi | Christian | 94.5 | 93.2 | 95.6 | 2596 |
| Burundi | Folk | 0.9 | 0.5 | 1.5 | 2596 |
| Burundi | Hindu | 0.0 | 0.0 | 0.0 | 2596 |
| Burundi | Muslim | 3.1 | 2.3 | 4.2 | 2596 |
| Burundi | Other | 0.0 | 0.0 | 0.0 | 2596 |
| Burundi | Unaffiliated | 1.5 | 1.1 | 2.2 | 2596 |
| CAR | Buddhist | 0.0 | 0.0 | 0.0 | 1688 |
| CAR | Christian | 90.7 | 88.1 | 92.7 | 1688 |
| CAR | Folk | 0.8 | 0.5 | 1.5 | 1688 |
| CAR | Hindu | 0.0 | 0.0 | 0.0 | 1688 |
| CAR | Muslim | 6.7 | 4.8 | 9.2 | 1688 |
| CAR | Other | 1.2 | 0.7 | 2.1 | 1688 |
| CAR | Unaffiliated | 0.6 | 0.3 | 1.3 | 1688 |
| Cambodia | Buddhist | 95.8 | 93.7 | 97.3 | 1440 |
| Cambodia | Christian | 0.9 | 0.4 | 2.0 | 1440 |
| Cambodia | Folk | 0.0 | 0.0 | 0.0 | 1440 |
| Cambodia | Hindu | 0.0 | 0.0 | 0.0 | 1440 |
| Cambodia | Muslim | 2.1 | 1.1 | 4.1 | 1440 |
| Cambodia | Other | 1.2 | 0.7 | 2.0 | 1440 |
| Cambodia | Unaffiliated | 0.0 | 0.0 | 0.0 | 1440 |
| Cameroon | Buddhist | 0.0 | 0.0 | 0.0 | 1824 |
| Cameroon | Christian | 95.9 | 94.1 | 97.1 | 1824 |
| Cameroon | Folk | 1.9 | 1.0 | 3.6 | 1824 |
| Cameroon | Hindu | 0.0 | 0.0 | 0.0 | 1824 |
| Cameroon | Muslim | 0.0 | 0.0 | 0.0 | 1824 |
| Cameroon | Other | 0.4 | 0.2 | 0.9 | 1824 |
| Cameroon | Unaffiliated | 1.8 | 1.2 | 2.8 | 1824 |
| Chad | Buddhist | 0.0 | 0.0 | 0.0 | 2858 |
| Chad | Christian | 48.1 | 44.5 | 51.7 | 2858 |
| Chad | Folk | 0.3 | 0.1 | 0.6 | 2858 |
| Chad | Hindu | 0.0 | 0.0 | 0.0 | 2858 |
| Chad | Muslim | 49.1 | 45.5 | 52.6 | 2858 |
| Chad | Other | 0.0 | 0.0 | 0.0 | 2858 |
| Chad | Unaffiliated | 2.5 | 1.8 | 3.5 | 2858 |
| Comoros | Buddhist | 0.0 | 0.0 | 0.0 | 629 |
| Comoros | Christian | 0.4 | 0.1 | 2.8 | 629 |
| Comoros | Folk | 0.0 | 0.0 | 0.0 | 629 |
| Comoros | Hindu | 0.0 | 0.0 | 0.0 | 629 |
| Comoros | Muslim | 99.6 | 97.2 | 99.9 | 629 |
| Comoros | Other | 0.0 | 0.0 | 0.0 | 629 |
| Comoros | Unaffiliated | 0.0 | 0.0 | 0.0 | 629 |
| Congo Brazzaville | Buddhist | 0.0 | 0.0 | 0.0 | 1768 |
| Congo Brazzaville | Christian | 76.3 | 72.9 | 79.4 | 1768 |
| Congo Brazzaville | Folk | 1.2 | 0.6 | 2.4 | 1768 |
| Congo Brazzaville | Hindu | 0.0 | 0.0 | 0.0 | 1768 |
| Congo Brazzaville | Muslim | 3.9 | 2.1 | 7.1 | 1768 |
| Congo Brazzaville | Other | 6.0 | 4.5 | 7.7 | 1768 |
| Congo Brazzaville | Unaffiliated | 12.7 | 10.8 | 14.9 | 1768 |
| Congo Democratic Republic | Buddhist | 0.0 | 0.0 | 0.0 | 4250 |
| Congo Democratic Republic | Christian | 85.3 | 83.2 | 87.1 | 4250 |
| Congo Democratic Republic | Folk | 5.9 | 4.5 | 7.6 | 4250 |
| Congo Democratic Republic | Hindu | 0.0 | 0.0 | 0.0 | 4250 |
| Congo Democratic Republic | Muslim | 2.6 | 1.8 | 3.6 | 4250 |
| Congo Democratic Republic | Other | 4.3 | 3.3 | 5.5 | 4250 |
| Congo Democratic Republic | Unaffiliated | 2.0 | 1.5 | 2.7 | 4250 |
| Costa Rica | Buddhist | 0.0 | 0.0 | 0.0 | 706 |
| Costa Rica | Christian | 93.3 | 90.4 | 95.4 | 706 |
| Costa Rica | Folk | 0.0 | 0.0 | 0.0 | 706 |
| Costa Rica | Hindu | 0.0 | 0.0 | 0.0 | 706 |
| Costa Rica | Muslim | 0.0 | 0.0 | 0.0 | 706 |
| Costa Rica | Other | 0.8 | 0.2 | 3.1 | 706 |
| Costa Rica | Unaffiliated | 5.9 | 4.0 | 8.6 | 706 |
| Côte d’Ivoire | Buddhist | 0.0 | 0.0 | 0.0 | 1784 |
| Côte d’Ivoire | Christian | 34.5 | 30.7 | 38.5 | 1784 |
| Côte d’Ivoire | Folk | 8.1 | 6.6 | 10.0 | 1784 |
| Côte d’Ivoire | Hindu | 0.0 | 0.0 | 0.0 | 1784 |
| Côte d’Ivoire | Muslim | 47.3 | 42.8 | 51.7 | 1784 |
| Côte d’Ivoire | Other | 0.7 | 0.4 | 1.4 | 1784 |
| Côte d’Ivoire | Unaffiliated | 9.4 | 7.6 | 11.6 | 1784 |
| Dominican Republic | Buddhist | 0.0 | 0.0 | 0.0 | 3939 |
| Dominican Republic | Christian | 76.7 | 74.8 | 78.5 | 3939 |
| Dominican Republic | Folk | 0.0 | 0.0 | 0.0 | 3939 |
| Dominican Republic | Hindu | 0.0 | 0.0 | 0.0 | 3939 |
| Dominican Republic | Muslim | 0.0 | 0.0 | 0.0 | 3939 |
| Dominican Republic | Other | 1.3 | 0.9 | 2.0 | 3939 |
| Dominican Republic | Unaffiliated | 22.0 | 20.2 | 23.8 | 3939 |
| Egypt | Buddhist | 0.0 | 0.0 | 0.0 | 3204 |
| Egypt | Christian | 3.4 | 2.7 | 4.3 | 3204 |
| Egypt | Folk | 0.0 | 0.0 | 0.0 | 3204 |
| Egypt | Hindu | 0.0 | 0.0 | 0.0 | 3204 |
| Egypt | Muslim | 96.6 | 95.7 | 97.3 | 3204 |
| Egypt | Other | 0.0 | 0.0 | 0.0 | 3204 |
| Egypt | Unaffiliated | 0.0 | 0.0 | 0.0 | 3204 |
| El Salvador | Buddhist | 0.0 | 0.0 | 0.0 | 1504 |
| El Salvador | Christian | 82.8 | 80.1 | 85.1 | 1504 |
| El Salvador | Folk | 0.0 | 0.0 | 0.0 | 1504 |
| El Salvador | Hindu | 0.0 | 0.0 | 0.0 | 1504 |
| El Salvador | Muslim | 0.0 | 0.0 | 0.0 | 1504 |
| El Salvador | Other | 0.5 | 0.2 | 1.4 | 1504 |
| El Salvador | Unaffiliated | 16.8 | 14.4 | 19.4 | 1504 |
| Eswatini | Buddhist | 0.0 | 0.0 | 0.0 | 538 |
| Eswatini | Christian | 94.6 | 92.2 | 96.3 | 538 |
| Eswatini | Folk | 1.8 | 1.0 | 3.2 | 538 |
| Eswatini | Hindu | 0.0 | 0.0 | 0.0 | 538 |
| Eswatini | Muslim | 0.0 | 0.0 | 0.0 | 538 |
| Eswatini | Other | 0.2 | 0.0 | 1.1 | 538 |
| Eswatini | Unaffiliated | 3.5 | 2.1 | 5.7 | 538 |
| Ethiopia | Buddhist | 0.0 | 0.0 | 0.0 | 1008 |
| Ethiopia | Christian | 63.6 | 54.4 | 71.8 | 1008 |
| Ethiopia | Folk | 2.1 | 0.6 | 6.9 | 1008 |
| Ethiopia | Hindu | 0.0 | 0.0 | 0.0 | 1008 |
| Ethiopia | Muslim | 34.1 | 25.5 | 43.9 | 1008 |
| Ethiopia | Other | 0.3 | 0.0 | 1.8 | 1008 |
| Ethiopia | Unaffiliated | 0.0 | 0.0 | 0.0 | 1008 |
| Gabon | Buddhist | 0.0 | 0.0 | 0.0 | 1196 |
| Gabon | Christian | 82.9 | 78.6 | 86.4 | 1196 |
| Gabon | Folk | 0.1 | 0.0 | 0.2 | 1196 |
| Gabon | Hindu | 0.0 | 0.0 | 0.0 | 1196 |
| Gabon | Muslim | 10.2 | 7.2 | 14.3 | 1196 |
| Gabon | Other | 0.3 | 0.1 | 0.7 | 1196 |
| Gabon | Unaffiliated | 6.6 | 5.0 | 8.6 | 1196 |
| Gambia | Buddhist | 0.0 | 0.0 | 0.0 | 1895 |
| Gambia | Christian | 1.9 | 1.2 | 3.1 | 1895 |
| Gambia | Folk | 0.0 | 0.0 | 0.0 | 1895 |
| Gambia | Hindu | 0.0 | 0.0 | 0.0 | 1895 |
| Gambia | Muslim | 98.1 | 96.9 | 98.8 | 1895 |
| Gambia | Other | 0.0 | 0.0 | 0.0 | 1895 |
| Gambia | Unaffiliated | 0.0 | 0.0 | 0.0 | 1895 |
| Ghana | Buddhist | 0.0 | 0.0 | 0.0 | 1679 |
| Ghana | Christian | 66.6 | 61.8 | 71.1 | 1679 |
| Ghana | Folk | 4.9 | 3.5 | 6.6 | 1679 |
| Ghana | Hindu | 0.0 | 0.0 | 0.0 | 1679 |
| Ghana | Muslim | 22.1 | 17.7 | 27.2 | 1679 |
| Ghana | Other | 0.5 | 0.1 | 1.5 | 1679 |
| Ghana | Unaffiliated | 6.0 | 4.5 | 7.9 | 1679 |
| Guatemala | Buddhist | 0.0 | 0.0 | 0.0 | 2408 |
| Guatemala | Christian | 88.3 | 86.5 | 89.9 | 2408 |
| Guatemala | Folk | 0.5 | 0.2 | 1.2 | 2408 |
| Guatemala | Hindu | 0.0 | 0.0 | 0.0 | 2408 |
| Guatemala | Muslim | 0.0 | 0.0 | 0.0 | 2408 |
| Guatemala | Other | 0.4 | 0.1 | 0.9 | 2408 |
| Guatemala | Unaffiliated | 10.8 | 9.3 | 12.4 | 2408 |
| Guinea | Buddhist | 0.0 | 0.0 | 0.0 | 1408 |
| Guinea | Christian | 10.3 | 8.0 | 13.3 | 1408 |
| Guinea | Folk | 0.2 | 0.0 | 1.3 | 1408 |
| Guinea | Hindu | 0.0 | 0.0 | 0.0 | 1408 |
| Guinea | Muslim | 87.8 | 84.9 | 90.3 | 1408 |
| Guinea | Other | 0.0 | 0.0 | 0.0 | 1408 |
| Guinea | Unaffiliated | 1.6 | 0.8 | 3.4 | 1408 |
| Guinea Bissau | Buddhist | 0.0 | 0.0 | 0.0 | 1409 |
| Guinea Bissau | Christian | 18.1 | 15.2 | 21.5 | 1409 |
| Guinea Bissau | Folk | 9.9 | 7.9 | 12.3 | 1409 |
| Guinea Bissau | Hindu | 0.0 | 0.0 | 0.0 | 1409 |
| Guinea Bissau | Muslim | 60.0 | 55.1 | 64.8 | 1409 |
| Guinea Bissau | Other | 0.8 | 0.4 | 1.5 | 1409 |
| Guinea Bissau | Unaffiliated | 11.2 | 9.1 | 13.6 | 1409 |
| Guyana | Buddhist | 0.0 | 0.0 | 0.0 | 686 |
| Guyana | Christian | 74.8 | 69.4 | 79.5 | 686 |
| Guyana | Folk | 0.0 | 0.0 | 0.0 | 686 |
| Guyana | Hindu | 15.9 | 12.2 | 20.4 | 686 |
| Guyana | Muslim | 6.8 | 4.2 | 10.8 | 686 |
| Guyana | Other | 1.2 | 0.3 | 5.2 | 686 |
| Guyana | Unaffiliated | 1.4 | 0.6 | 3.1 | 686 |
| Haiti | Buddhist | 0.0 | 0.0 | 0.0 | 1196 |
| Haiti | Christian | 87.1 | 83.9 | 89.7 | 1196 |
| Haiti | Folk | 1.3 | 0.7 | 2.4 | 1196 |
| Haiti | Hindu | 0.0 | 0.0 | 0.0 | 1196 |
| Haiti | Muslim | 0.0 | 0.0 | 0.0 | 1196 |
| Haiti | Other | 0.0 | 0.0 | 0.0 | 1196 |
| Haiti | Unaffiliated | 11.6 | 9.1 | 14.7 | 1196 |
| Honduras | Buddhist | 0.0 | 0.0 | 0.0 | 2275 |
| Honduras | Christian | 85.7 | 83.6 | 87.5 | 2275 |
| Honduras | Folk | 0.0 | 0.0 | 0.0 | 2275 |
| Honduras | Hindu | 0.0 | 0.0 | 0.0 | 2275 |
| Honduras | Muslim | 0.0 | 0.0 | 0.0 | 2275 |
| Honduras | Other | 0.7 | 0.4 | 1.4 | 2275 |
| Honduras | Unaffiliated | 13.6 | 11.8 | 15.7 | 2275 |
| India | Buddhist | 0.7 | 0.6 | 0.9 | 49284 |
| India | Christian | 2.1 | 1.9 | 2.3 | 49284 |
| India | Folk | 0.0 | 0.0 | 0.0 | 49284 |
| India | Hindu | 78.3 | 77.6 | 79.1 | 49284 |
| India | Muslim | 16.9 | 16.2 | 17.6 | 49284 |
| India | Other | 1.9 | 1.8 | 2.1 | 49284 |
| India | Unaffiliated | 0.0 | 0.0 | 0.1 | 49284 |
| Iraq | Buddhist | 0.0 | 0.0 | 0.0 | 3205 |
| Iraq | Christian | 0.4 | 0.2 | 0.6 | 3205 |
| Iraq | Folk | 0.1 | 0.0 | 0.6 | 3205 |
| Iraq | Hindu | 0.0 | 0.0 | 0.0 | 3205 |
| Iraq | Muslim | 99.6 | 99.2 | 99.8 | 3205 |
| Iraq | Other | 0.0 | 0.0 | 0.0 | 3205 |
| Iraq | Unaffiliated | 0.0 | 0.0 | 0.0 | 3205 |
| Jamaica | Buddhist | 0.0 | 0.0 | 0.0 | 314 |
| Jamaica | Christian | 82.9 | 76.5 | 87.9 | 314 |
| Jamaica | Folk | 0.8 | 0.2 | 2.9 | 314 |
| Jamaica | Hindu | 0.0 | 0.0 | 0.0 | 314 |
| Jamaica | Muslim | 0.0 | 0.0 | 0.0 | 314 |
| Jamaica | Other | 1.4 | 0.2 | 8.2 | 314 |
| Jamaica | Unaffiliated | 14.9 | 10.1 | 21.3 | 314 |
| Kazakhstan | Buddhist | 0.0 | 0.0 | 0.0 | 1084 |
| Kazakhstan | Christian | 18.5 | 15.8 | 21.4 | 1084 |
| Kazakhstan | Folk | 0.0 | 0.0 | 0.0 | 1084 |
| Kazakhstan | Hindu | 0.0 | 0.0 | 0.0 | 1084 |
| Kazakhstan | Muslim | 78.4 | 75.3 | 81.1 | 1084 |
| Kazakhstan | Other | 0.0 | 0.0 | 0.0 | 1084 |
| Kazakhstan | Unaffiliated | 3.2 | 2.2 | 4.6 | 1084 |
| Kenya | Buddhist | 0.0 | 0.0 | 0.0 | 4045 |
| Kenya | Christian | 88.5 | 87.1 | 89.8 | 4045 |
| Kenya | Folk | 0.0 | 0.0 | 0.0 | 4045 |
| Kenya | Hindu | 0.0 | 0.0 | 0.0 | 4045 |
| Kenya | Muslim | 8.6 | 7.5 | 10.0 | 4045 |
| Kenya | Other | 0.1 | 0.0 | 0.3 | 4045 |
| Kenya | Unaffiliated | 2.7 | 2.1 | 3.5 | 4045 |
| Kiribati | Buddhist | 0.0 | 0.0 | 0.0 | 453 |
| Kiribati | Christian | 94.0 | 91.0 | 96.0 | 453 |
| Kiribati | Folk | 0.0 | 0.0 | 0.0 | 453 |
| Kiribati | Hindu | 0.0 | 0.0 | 0.0 | 453 |
| Kiribati | Muslim | 0.0 | 0.0 | 0.0 | 453 |
| Kiribati | Other | 5.7 | 3.8 | 8.5 | 453 |
| Kiribati | Unaffiliated | 0.3 | 0.0 | 2.1 | 453 |
| Kosovo | Buddhist | 0.0 | 0.0 | 0.0 | 305 |
| Kosovo | Christian | 2.4 | 1.1 | 4.9 | 305 |
| Kosovo | Folk | 0.0 | 0.0 | 0.0 | 305 |
| Kosovo | Hindu | 0.0 | 0.0 | 0.0 | 305 |
| Kosovo | Muslim | 97.6 | 95.1 | 98.9 | 305 |
| Kosovo | Other | 0.0 | 0.0 | 0.0 | 305 |
| Kosovo | Unaffiliated | 0.0 | 0.0 | 0.0 | 305 |
| Kyrgyzstan | Buddhist | 0.0 | 0.0 | 0.0 | 879 |
| Kyrgyzstan | Christian | 3.0 | 1.7 | 5.2 | 879 |
| Kyrgyzstan | Folk | 0.0 | 0.0 | 0.0 | 879 |
| Kyrgyzstan | Hindu | 0.0 | 0.0 | 0.0 | 879 |
| Kyrgyzstan | Muslim | 96.1 | 93.5 | 97.7 | 879 |
| Kyrgyzstan | Other | 0.2 | 0.0 | 1.6 | 879 |
| Kyrgyzstan | Unaffiliated | 0.7 | 0.2 | 3.0 | 879 |
| Lao | Buddhist | 57.9 | 54.3 | 61.4 | 2214 |
| Lao | Christian | 2.2 | 1.4 | 3.3 | 2214 |
| Lao | Folk | 39.4 | 35.9 | 43.0 | 2214 |
| Lao | Hindu | 0.0 | 0.0 | 0.0 | 2214 |
| Lao | Muslim | 0.0 | 0.0 | 0.0 | 2214 |
| Lao | Other | 0.5 | 0.2 | 1.0 | 2214 |
| Lao | Unaffiliated | 0.1 | 0.0 | 0.5 | 2214 |
| Liberia | Buddhist | 0.0 | 0.0 | 0.0 | 1063 |
| Liberia | Christian | 82.2 | 77.7 | 86.0 | 1063 |
| Liberia | Folk | 1.0 | 0.4 | 2.6 | 1063 |
| Liberia | Hindu | 0.0 | 0.0 | 0.0 | 1063 |
| Liberia | Muslim | 14.6 | 11.0 | 19.2 | 1063 |
| Liberia | Other | 0.0 | 0.0 | 0.0 | 1063 |
| Liberia | Unaffiliated | 2.2 | 1.2 | 3.8 | 1063 |
| Madagascar | Buddhist | 0.0 | 0.0 | 0.0 | 2590 |
| Madagascar | Christian | 60.0 | 57.2 | 62.8 | 2590 |
| Madagascar | Folk | 5.0 | 3.8 | 6.4 | 2590 |
| Madagascar | Hindu | 0.0 | 0.0 | 0.0 | 2590 |
| Madagascar | Muslim | 0.6 | 0.4 | 1.0 | 2590 |
| Madagascar | Other | 7.5 | 6.2 | 9.0 | 2590 |
| Madagascar | Unaffiliated | 26.9 | 24.5 | 29.4 | 2590 |
| Malawi | Buddhist | 0.0 | 0.0 | 0.0 | 3248 |
| Malawi | Christian | 82.6 | 80.1 | 84.8 | 3248 |
| Malawi | Folk | 0.0 | 0.0 | 0.0 | 3248 |
| Malawi | Hindu | 0.0 | 0.0 | 0.0 | 3248 |
| Malawi | Muslim | 16.7 | 14.5 | 19.2 | 3248 |
| Malawi | Other | 0.2 | 0.1 | 0.4 | 3248 |
| Malawi | Unaffiliated | 0.5 | 0.3 | 1.0 | 3248 |
| Mali | Buddhist | 0.0 | 0.0 | 0.0 | 1946 |
| Mali | Christian | 2.5 | 1.6 | 4.0 | 1946 |
| Mali | Folk | 0.3 | 0.1 | 0.9 | 1946 |
| Mali | Hindu | 0.0 | 0.0 | 0.0 | 1946 |
| Mali | Muslim | 93.4 | 90.9 | 95.2 | 1946 |
| Mali | Other | 0.0 | 0.0 | 0.0 | 1946 |
| Mali | Unaffiliated | 3.8 | 2.4 | 5.8 | 1946 |
| Mongolia | Buddhist | 45.2 | 40.8 | 49.6 | 1075 |
| Mongolia | Christian | 0.7 | 0.3 | 1.5 | 1075 |
| Mongolia | Folk | 3.3 | 2.0 | 5.4 | 1075 |
| Mongolia | Hindu | 0.0 | 0.0 | 0.0 | 1075 |
| Mongolia | Muslim | 4.6 | 3.5 | 6.0 | 1075 |
| Mongolia | Other | 0.0 | 0.0 | 0.1 | 1075 |
| Mongolia | Unaffiliated | 46.2 | 41.6 | 50.9 | 1075 |
| Montenegro | Buddhist | 0.0 | 0.0 | 0.0 | 263 |
| Montenegro | Christian | 68.9 | 57.4 | 78.5 | 263 |
| Montenegro | Folk | 0.0 | 0.0 | 0.0 | 263 |
| Montenegro | Hindu | 0.0 | 0.0 | 0.0 | 263 |
| Montenegro | Muslim | 27.4 | 18.2 | 38.9 | 263 |
| Montenegro | Other | 3.1 | 1.0 | 9.2 | 263 |
| Montenegro | Unaffiliated | 0.6 | 0.1 | 3.0 | 263 |
| Mozambique | Buddhist | 0.0 | 0.0 | 0.0 | 1028 |
| Mozambique | Christian | 56.4 | 51.4 | 61.2 | 1028 |
| Mozambique | Folk | 13.7 | 10.8 | 17.3 | 1028 |
| Mozambique | Hindu | 0.0 | 0.0 | 0.0 | 1028 |
| Mozambique | Muslim | 19.8 | 16.4 | 23.8 | 1028 |
| Mozambique | Other | 2.4 | 1.4 | 4.0 | 1028 |
| Mozambique | Unaffiliated | 7.7 | 5.6 | 10.5 | 1028 |
| Namibia | Buddhist | 0.0 | 0.0 | 0.0 | 990 |
| Namibia | Christian | 89.2 | 86.2 | 91.6 | 990 |
| Namibia | Folk | 0.0 | 0.0 | 0.0 | 990 |
| Namibia | Hindu | 0.0 | 0.0 | 0.0 | 990 |
| Namibia | Muslim | 0.0 | 0.0 | 0.0 | 990 |
| Namibia | Other | 8.6 | 6.6 | 11.3 | 990 |
| Namibia | Unaffiliated | 2.1 | 1.3 | 3.6 | 990 |
| Nepal | Buddhist | 6.2 | 4.6 | 8.4 | 1327 |
| Nepal | Christian | 2.1 | 1.3 | 3.5 | 1327 |
| Nepal | Folk | 2.5 | 1.3 | 4.6 | 1327 |
| Nepal | Hindu | 83.0 | 79.2 | 86.3 | 1327 |
| Nepal | Muslim | 6.0 | 3.8 | 9.5 | 1327 |
| Nepal | Other | 0.1 | 0.0 | 0.5 | 1327 |
| Nepal | Unaffiliated | 0.0 | 0.0 | 0.0 | 1327 |
| Nigeria | Buddhist | 0.0 | 0.0 | 0.0 | 6059 |
| Nigeria | Christian | 38.2 | 36.1 | 40.3 | 6059 |
| Nigeria | Folk | 0.2 | 0.1 | 0.5 | 6059 |
| Nigeria | Hindu | 0.0 | 0.0 | 0.0 | 6059 |
| Nigeria | Muslim | 61.3 | 59.2 | 63.4 | 6059 |
| Nigeria | Other | 0.2 | 0.2 | 0.4 | 6059 |
| Nigeria | Unaffiliated | 0.0 | 0.0 | 0.0 | 6059 |
| North Macedonia | Buddhist | 0.0 | 0.0 | 0.0 | 307 |
| North Macedonia | Christian | 55.0 | 43.0 | 66.5 | 307 |
| North Macedonia | Folk | 0.0 | 0.0 | 0.0 | 307 |
| North Macedonia | Hindu | 0.0 | 0.0 | 0.0 | 307 |
| North Macedonia | Muslim | 45.0 | 33.5 | 57.0 | 307 |
| North Macedonia | Other | 0.0 | 0.0 | 0.0 | 307 |
| North Macedonia | Unaffiliated | 0.0 | 0.0 | 0.0 | 307 |
| Papua New Guinea | Buddhist | 0.0 | 0.0 | 0.0 | 1813 |
| Papua New Guinea | Christian | 99.0 | 97.9 | 99.5 | 1813 |
| Papua New Guinea | Folk | 0.0 | 0.0 | 0.0 | 1813 |
| Papua New Guinea | Hindu | 0.0 | 0.0 | 0.0 | 1813 |
| Papua New Guinea | Muslim | 0.0 | 0.0 | 0.0 | 1813 |
| Papua New Guinea | Other | 0.5 | 0.2 | 1.3 | 1813 |
| Papua New Guinea | Unaffiliated | 0.5 | 0.1 | 1.6 | 1813 |
| Paraguay | Buddhist | 0.0 | 0.0 | 0.0 | 1011 |
| Paraguay | Christian | 95.0 | 92.7 | 96.7 | 1011 |
| Paraguay | Folk | 0.0 | 0.0 | 0.0 | 1011 |
| Paraguay | Hindu | 0.0 | 0.0 | 0.0 | 1011 |
| Paraguay | Muslim | 0.0 | 0.0 | 0.0 | 1011 |
| Paraguay | Other | 1.8 | 0.8 | 3.7 | 1011 |
| Paraguay | Unaffiliated | 3.2 | 2.0 | 5.0 | 1011 |
| Philippines | Buddhist | 0.0 | 0.0 | 0.0 | 1986 |
| Philippines | Christian | 92.7 | 90.9 | 94.2 | 1986 |
| Philippines | Folk | 0.0 | 0.0 | 0.0 | 1986 |
| Philippines | Hindu | 0.0 | 0.0 | 0.0 | 1986 |
| Philippines | Muslim | 6.2 | 4.8 | 7.9 | 1986 |
| Philippines | Other | 1.0 | 0.6 | 1.7 | 1986 |
| Philippines | Unaffiliated | 0.1 | 0.0 | 0.3 | 1986 |
| Rwanda | Buddhist | 0.0 | 0.0 | 0.0 | 1535 |
| Rwanda | Christian | 97.5 | 96.6 | 98.2 | 1535 |
| Rwanda | Folk | 0.0 | 0.0 | 0.0 | 1535 |
| Rwanda | Hindu | 0.0 | 0.0 | 0.0 | 1535 |
| Rwanda | Muslim | 1.9 | 1.3 | 2.8 | 1535 |
| Rwanda | Other | 0.1 | 0.0 | 0.6 | 1535 |
| Rwanda | Unaffiliated | 0.5 | 0.2 | 1.0 | 1535 |
| São Tomé and Príncipe | Buddhist | 0.0 | 0.0 | 0.0 | 349 |
| São Tomé and Príncipe | Christian | 79.0 | 73.2 | 83.8 | 349 |
| São Tomé and Príncipe | Folk | 0.0 | 0.0 | 0.0 | 349 |
| São Tomé and Príncipe | Hindu | 0.0 | 0.0 | 0.0 | 349 |
| São Tomé and Príncipe | Muslim | 0.0 | 0.0 | 0.0 | 349 |
| São Tomé and Príncipe | Other | 7.2 | 4.8 | 10.8 | 349 |
| São Tomé and Príncipe | Unaffiliated | 13.8 | 9.5 | 19.5 | 349 |
| Senegal | Buddhist | 0.0 | 0.0 | 0.0 | 1183 |
| Senegal | Christian | 2.6 | 1.5 | 4.7 | 1183 |
| Senegal | Folk | 0.0 | 0.0 | 0.0 | 1183 |
| Senegal | Hindu | 0.0 | 0.0 | 0.0 | 1183 |
| Senegal | Muslim | 97.4 | 95.3 | 98.5 | 1183 |
| Senegal | Other | 0.0 | 0.0 | 0.0 | 1183 |
| Senegal | Unaffiliated | 0.0 | 0.0 | 0.0 | 1183 |
| Serbia | Buddhist | 0.0 | 0.0 | 0.0 | 382 |
| Serbia | Christian | 94.0 | 89.3 | 96.7 | 382 |
| Serbia | Folk | 0.0 | 0.0 | 0.0 | 382 |
| Serbia | Hindu | 0.0 | 0.0 | 0.0 | 382 |
| Serbia | Muslim | 4.1 | 1.8 | 9.0 | 382 |
| Serbia | Other | 0.4 | 0.1 | 1.7 | 382 |
| Serbia | Unaffiliated | 1.5 | 0.7 | 3.4 | 382 |
| Sierra Leone | Buddhist | 0.0 | 0.0 | 0.0 | 1861 |
| Sierra Leone | Christian | 20.5 | 17.8 | 23.5 | 1861 |
| Sierra Leone | Folk | 0.0 | 0.0 | 0.0 | 1861 |
| Sierra Leone | Hindu | 0.0 | 0.0 | 0.0 | 1861 |
| Sierra Leone | Muslim | 79.5 | 76.5 | 82.2 | 1861 |
| Sierra Leone | Other | 0.0 | 0.0 | 0.0 | 1861 |
| Sierra Leone | Unaffiliated | 0.0 | 0.0 | 0.2 | 1861 |
| Suriname | Buddhist | 0.0 | 0.0 | 0.0 | 755 |
| Suriname | Christian | 62.3 | 56.9 | 67.4 | 755 |
| Suriname | Folk | 2.4 | 1.4 | 4.2 | 755 |
| Suriname | Hindu | 14.1 | 11.0 | 17.8 | 755 |
| Suriname | Muslim | 11.4 | 8.1 | 16.0 | 755 |
| Suriname | Other | 1.5 | 0.6 | 3.5 | 755 |
| Suriname | Unaffiliated | 8.3 | 6.0 | 11.5 | 755 |
| Thailand | Buddhist | 91.2 | 88.7 | 93.2 | 2879 |
| Thailand | Christian | 1.0 | 0.6 | 1.9 | 2879 |
| Thailand | Folk | 0.0 | 0.0 | 0.0 | 2879 |
| Thailand | Hindu | 0.0 | 0.0 | 0.0 | 2879 |
| Thailand | Muslim | 7.7 | 5.8 | 10.1 | 2879 |
| Thailand | Other | 0.0 | 0.0 | 0.0 | 2879 |
| Thailand | Unaffiliated | 0.1 | 0.0 | 0.4 | 2879 |
| Timor-Leste | Buddhist | 0.0 | 0.0 | 0.0 | 1423 |
| Timor-Leste | Christian | 99.8 | 99.4 | 99.9 | 1423 |
| Timor-Leste | Folk | 0.0 | 0.0 | 0.0 | 1423 |
| Timor-Leste | Hindu | 0.0 | 0.0 | 0.0 | 1423 |
| Timor-Leste | Muslim | 0.2 | 0.1 | 0.6 | 1423 |
| Timor-Leste | Other | 0.0 | 0.0 | 0.0 | 1423 |
| Timor-Leste | Unaffiliated | 0.0 | 0.0 | 0.0 | 1423 |
| Togo | Buddhist | 0.0 | 0.0 | 0.0 | 973 |
| Togo | Christian | 47.4 | 42.9 | 52.0 | 973 |
| Togo | Folk | 22.0 | 18.4 | 26.2 | 973 |
| Togo | Hindu | 0.0 | 0.0 | 0.0 | 973 |
| Togo | Muslim | 22.4 | 17.9 | 27.7 | 973 |
| Togo | Other | 2.1 | 1.2 | 3.8 | 973 |
| Togo | Unaffiliated | 6.0 | 4.3 | 8.2 | 973 |
| Tonga | Buddhist | 0.0 | 0.0 | 0.0 | 246 |
| Tonga | Christian | 98.2 | 96.1 | 99.2 | 246 |
| Tonga | Folk | 0.0 | 0.0 | 0.0 | 246 |
| Tonga | Hindu | 0.0 | 0.0 | 0.0 | 246 |
| Tonga | Muslim | 0.0 | 0.0 | 0.0 | 246 |
| Tonga | Other | 1.4 | 0.6 | 3.4 | 246 |
| Tonga | Unaffiliated | 0.3 | 0.0 | 2.3 | 246 |
| Uganda | Buddhist | 0.0 | 0.0 | 0.0 | 2922 |
| Uganda | Christian | 83.9 | 81.4 | 86.1 | 2922 |
| Uganda | Folk | 0.0 | 0.0 | 0.1 | 2922 |
| Uganda | Hindu | 0.0 | 0.0 | 0.0 | 2922 |
| Uganda | Muslim | 15.2 | 13.0 | 17.7 | 2922 |
| Uganda | Other | 0.8 | 0.5 | 1.3 | 2922 |
| Uganda | Unaffiliated | 0.1 | 0.0 | 0.4 | 2922 |
| Vietnam | Buddhist | 14.2 | 11.4 | 17.6 | 785 |
| Vietnam | Christian | 8.3 | 6.2 | 11.2 | 785 |
| Vietnam | Folk | 0.0 | 0.0 | 0.0 | 785 |
| Vietnam | Hindu | 0.0 | 0.0 | 0.0 | 785 |
| Vietnam | Muslim | 0.2 | 0.0 | 1.5 | 785 |
| Vietnam | Other | 1.6 | 0.8 | 3.5 | 785 |
| Vietnam | Unaffiliated | 75.6 | 71.4 | 79.3 | 785 |
| Zambia | Buddhist | 0.0 | 0.0 | 0.0 | 1928 |
| Zambia | Christian | 98.8 | 97.8 | 99.3 | 1928 |
| Zambia | Folk | 0.0 | 0.0 | 0.0 | 1928 |
| Zambia | Hindu | 0.0 | 0.0 | 0.0 | 1928 |
| Zambia | Muslim | 0.5 | 0.2 | 1.4 | 1928 |
| Zambia | Other | 0.8 | 0.4 | 1.5 | 1928 |
| Zambia | Unaffiliated | 0.0 | 0.0 | 0.0 | 1928 |
| Zimbabwe | Buddhist | 0.0 | 0.0 | 0.0 | 1153 |
| Zimbabwe | Christian | 70.2 | 67.1 | 73.2 | 1153 |
| Zimbabwe | Folk | 12.5 | 10.6 | 14.6 | 1153 |
| Zimbabwe | Hindu | 0.0 | 0.0 | 0.0 | 1153 |
| Zimbabwe | Muslim | 0.7 | 0.3 | 1.4 | 1153 |
| Zimbabwe | Other | 0.2 | 0.1 | 0.8 | 1153 |
| Zimbabwe | Unaffiliated | 16.4 | 14.1 | 19.0 | 1153 |

Supplementary Table 2. National prevalence of no-DPT

| Country | No-DPT prevalence | 95% Confidence interval | | N |
| --- | --- | --- | --- | --- |
|  |  | lower limit | upper limit |  |
| Angola | 31.2 | 28.6 | 34.0 | 2845 |
| Bangladesh | 1.5 | 0.9 | 2.5 | 1666 |
| Belize | 7.1 | 4.4 | 11.3 | 502 |
| Benin | 15.8 | 13.8 | 17.9 | 2522 |
| Burkina Faso | 5.6 | 4.4 | 7.0 | 2777 |
| Burundi | 0.8 | 0.5 | 1.2 | 2596 |
| CAR | 45.0 | 41.7 | 48.3 | 1688 |
| Cambodia | 6.1 | 4.7 | 7.9 | 1440 |
| Cameroon | 16.7 | 14.2 | 19.5 | 1824 |
| Chad | 41.8 | 38.8 | 44.9 | 2858 |
| Comoros | 17.8 | 14.1 | 22.3 | 629 |
| Congo Brazzaville | 14.0 | 12.0 | 16.3 | 1768 |
| Congo Democratic Republic | 34.1 | 30.2 | 38.2 | 4250 |
| Costa Rica | 2.3 | 1.1 | 4.7 | 706 |
| Côte d’Ivoire | 19.6 | 17.3 | 22.2 | 1784 |
| Dominican Republic | 8.9 | 7.7 | 10.2 | 3939 |
| Egypt | 0.6 | 0.3 | 1.1 | 3204 |
| El Salvador | 1.1 | 0.5 | 2.5 | 1504 |
| Eswatini | 3.4 | 2.1 | 5.6 | 538 |
| Ethiopia | 23.7 | 19.2 | 28.9 | 1008 |
| Gabon | 11.6 | 9.0 | 14.7 | 1196 |
| Gambia | 3.2 | 2.2 | 4.7 | 1895 |
| Ghana | 4.0 | 2.8 | 5.8 | 1679 |
| Guatemala | 2.5 | 1.8 | 3.3 | 2408 |
| Guinea | 37.7 | 34.1 | 41.4 | 1408 |
| Guinea Bissau | 7.0 | 5.2 | 9.4 | 1409 |
| Guyana | 4.5 | 2.7 | 7.2 | 686 |
| Haiti | 16.5 | 13.3 | 20.1 | 1196 |
| Honduras | 0.9 | 0.5 | 1.5 | 2275 |
| India | 10.5 | 10.1 | 10.9 | 49284 |
| Iraq | 13.3 | 11.4 | 15.5 | 3205 |
| Jamaica | 4.8 | 2.5 | 9.3 | 314 |
| Kazakhstan | 1.2 | 0.6 | 2.2 | 1084 |
| Kenya | 2.5 | 1.9 | 3.3 | 4045 |
| Kiribati | 40.1 | 35.1 | 45.4 | 453 |
| Kosovo | 2.7 | 1.4 | 5.2 | 305 |
| Kyrgyzstan | 2.1 | 1.2 | 3.6 | 879 |
| Lao | 27.1 | 24.7 | 29.7 | 2214 |
| Liberia | 8.6 | 6.3 | 11.5 | 1063 |
| Madagascar | 28.5 | 25.9 | 31.2 | 2590 |
| Malawi | 2.6 | 1.9 | 3.5 | 3248 |
| Mali | 17.9 | 15.1 | 21.1 | 1946 |
| Mongolia | 3.0 | 1.9 | 4.5 | 1075 |
| Montenegro | 5.6 | 2.7 | 11.1 | 263 |
| Mozambique | 10.0 | 6.8 | 14.4 | 1028 |
| Namibia | 7.3 | 5.5 | 9.7 | 990 |
| Nepal | 10.5 | 8.5 | 13.0 | 1327 |
| Nigeria | 34.7 | 32.7 | 36.7 | 6059 |
| North Macedonia | 4.1 | 1.7 | 9.5 | 307 |
| Papua New Guinea | 36.1 | 32.2 | 40.2 | 1813 |
| Paraguay | 5.2 | 3.9 | 6.9 | 1011 |
| Philippines | 13.4 | 11.5 | 15.5 | 1986 |
| Rwanda | 0.9 | 0.5 | 1.7 | 1535 |
| São Tomé and Príncipe | 2.3 | 1.1 | 4.7 | 349 |
| Senegal | 3.8 | 2.6 | 5.6 | 1183 |
| Serbia | 3.5 | 1.9 | 6.7 | 382 |
| Sierra Leone | 5.4 | 4.2 | 6.9 | 1861 |
| Suriname | 20.0 | 15.8 | 25.1 | 755 |
| Thailand | 3.1 | 1.8 | 5.4 | 2879 |
| Timor-Leste | 21.6 | 18.8 | 24.7 | 1423 |
| Togo | 9.2 | 6.8 | 12.2 | 973 |
| Tonga | 3.6 | 1.4 | 8.8 | 246 |
| Uganda | 5.1 | 4.1 | 6.3 | 2922 |
| Vietnam | 3.8 | 2.6 | 5.5 | 785 |
| Zambia | 2.1 | 1.4 | 3.1 | 1928 |
| Zimbabwe | 5.5 | 3.9 | 7.6 | 1153 |

Supplementary Table 3. No-DPT prevalence according to religious group

| Country | Year | Religious group | No-DPT prevalence | N |
| --- | --- | --- | --- | --- |
| Angola | 2015 | Muslim | 44.3 | 4 |
| Angola | 2015 | Christian | 29.9 | 887 |
| Angola | 2015 | Folk | 38.6 | 5 |
| Angola | 2015 | Unaffiliated | 51.0 | 89 |
| Angola | 2015 | Other | 37.5 | 4 |
| Bangladesh | 2017 | Muslim | 1.6 | 27 |
| Bangladesh | 2017 | Christian | 0.0 | 0 |
| Bangladesh | 2017 | Hindu | 0.8 | 1 |
| Bangladesh | 2017 | Buddhist | 0.0 | 0 |
| Belize | 2015 | Christian | 4.3 | 12 |
| Belize | 2015 | Unaffiliated | 9.1 | 11 |
| Belize | 2015 | Other | 14.4 | 7 |
| Benin | 2017 | Muslim | 24.5 | 217 |
| Benin | 2017 | Christian | 9.7 | 113 |
| Benin | 2017 | Folk | 14.7 | 40 |
| Benin | 2017 | Unaffiliated | 16.4 | 30 |
| Benin | 2017 | Other | 23.6 | 9 |
| Burkina Faso | 2010 | Muslim | 5.6 | 101 |
| Burkina Faso | 2010 | Christian | 4.5 | 31 |
| Burkina Faso | 2010 | Folk | 9.3 | 22 |
| Burkina Faso | 2010 | Unaffiliated | 0.0 | 0 |
| Burkina Faso | 2010 | Other | 0.0 | 0 |
| Burundi | 2016 | Muslim | 0.4 | 1 |
| Burundi | 2016 | Christian | 0.7 | 16 |
| Burundi | 2016 | Folk | 0.0 | 0 |
| Burundi | 2016 | Unaffiliated | 6.7 | 2 |
| CAR | 2018 | Muslim | 40.3 | 91 |
| CAR | 2018 | Christian | 45.3 | 614 |
| CAR | 2018 | Folk | 62.0 | 6 |
| CAR | 2018 | Unaffiliated | 50.9 | 5 |
| CAR | 2018 | Other | 32.8 | 7 |
| Cambodia | 2014 | Muslim | 21.5 | 4 |
| Cambodia | 2014 | Christian | 30.0 | 2 |
| Cambodia | 2014 | Other | 21.5 | 10 |
| Cambodia | 2014 | Buddhist | 5.3 | 65 |
| Cameroon | 2018 | Christian | 16.1 | 284 |
| Cameroon | 2018 | Folk | 39.1 | 10 |
| Cameroon | 2018 | Unaffiliated | 27.0 | 10 |
| Cameroon | 2018 | Other | 0.0 | 0 |
| Chad | 2014 | Muslim | 58.1 | 1058 |
| Chad | 2014 | Christian | 25.3 | 262 |
| Chad | 2014 | Folk | 88.6 | 8 |
| Chad | 2014 | Unaffiliated | 33.7 | 21 |
| Comoros | 2012 | Muslim | 17.5 | 106 |
| Comoros | 2012 | Christian | 96.2 | 1 |
| Congo Brazzaville | 2014 | Muslim | 4.3 | 9 |
| Congo Brazzaville | 2014 | Christian | 12.7 | 252 |
| Congo Brazzaville | 2014 | Folk | 9.2 | 3 |
| Congo Brazzaville | 2014 | Unaffiliated | 25.6 | 102 |
| Congo Brazzaville | 2014 | Other | 13.7 | 23 |
| Congo Democratic Republic | 2017 | Muslim | 43.0 | 62 |
| Congo Democratic Republic | 2017 | Christian | 32.3 | 1488 |
| Congo Democratic Republic | 2017 | Folk | 43.9 | 143 |
| Congo Democratic Republic | 2017 | Unaffiliated | 59.2 | 58 |
| Congo Democratic Republic | 2017 | Other | 38.0 | 86 |
| Costa Rica | 2018 | Christian | 2.3 | 21 |
| Costa Rica | 2018 | Unaffiliated | 3.4 | 4 |
| Costa Rica | 2018 | Other | 0.0 | 0 |
| Côte d’Ivoire | 2016 | Muslim | 23.0 | 217 |
| Côte d’Ivoire | 2016 | Christian | 11.7 | 75 |
| Côte d’Ivoire | 2016 | Folk | 36.6 | 73 |
| Côte d’Ivoire | 2016 | Unaffiliated | 18.2 | 37 |
| Côte d’Ivoire | 2016 | Other | 2.9 | 1 |
| Dominican Republic | 2014 | Christian | 8.9 | 272 |
| Dominican Republic | 2014 | Unaffiliated | 8.7 | 84 |
| Dominican Republic | 2014 | Other | 11.1 | 5 |
| Egypt | 2014 | Muslim | 0.6 | 24 |
| Egypt | 2014 | Christian | 0.0 | 0 |
| El Salvador | 2014 | Christian | 0.9 | 8 |
| El Salvador | 2014 | Unaffiliated | 2.3 | 3 |
| El Salvador | 2014 | Other | 0.0 | 0 |
| Eswatini | 2014 | Christian | 3.5 | 16 |
| Eswatini | 2014 | Folk | 0.0 | 0 |
| Eswatini | 2014 | Unaffiliated | 4.5 | 1 |
| Eswatini | 2014 | Other | 0.0 | 0 |
| Ethiopia | 2019 | Muslim | 31.2 | 177 |
| Ethiopia | 2019 | Christian | 19.2 | 95 |
| Ethiopia | 2019 | Folk | 35.4 | 7 |
| Ethiopia | 2019 | Other | 48.3 | 2 |
| Gabon | 2012 | Muslim | 10.1 | 7 |
| Gabon | 2012 | Christian | 11.5 | 147 |
| Gabon | 2012 | Folk | 0.0 | 0 |
| Gabon | 2012 | Unaffiliated | 13.0 | 26 |
| Gabon | 2012 | Other | 52.4 | 2 |
| Gambia | 2018 | Muslim | 3.2 | 53 |
| Gambia | 2018 | Christian | 5.7 | 1 |
| Ghana | 2017 | Muslim | 3.9 | 17 |
| Ghana | 2017 | Christian | 4.0 | 44 |
| Ghana | 2017 | Folk | 4.2 | 8 |
| Ghana | 2017 | Unaffiliated | 4.8 | 6 |
| Ghana | 2017 | Other | 0.0 | 0 |
| Guatemala | 2014 | Christian | 2.3 | 45 |
| Guatemala | 2014 | Folk | 10.1 | 1 |
| Guatemala | 2014 | Unaffiliated | 3.6 | 8 |
| Guatemala | 2014 | Other | 0.0 | 0 |
| Guinea | 2018 | Muslim | 39.1 | 513 |
| Guinea | 2018 | Christian | 31.0 | 37 |
| Guinea | 2018 | Folk | 0.0 | 0 |
| Guinea | 2018 | Unaffiliated | 8.5 | 1 |
| Guinea Bissau | 2018 | Muslim | 7.4 | 58 |
| Guinea Bissau | 2018 | Christian | 8.8 | 18 |
| Guinea Bissau | 2018 | Folk | 6.9 | 18 |
| Guinea Bissau | 2018 | Unaffiliated | 3.1 | 10 |
| Guinea Bissau | 2018 | Other | 0.0 | 0 |
| Guyana | 2014 | Muslim | 3.9 | 2 |
| Guyana | 2014 | Christian | 5.2 | 30 |
| Guyana | 2014 | Hindu | 1.4 | 1 |
| Guyana | 2014 | Unaffiliated | 1.4 | 1 |
| Guyana | 2014 | Other | 7.2 | 2 |
| Haiti | 2016 | Christian | 15.9 | 157 |
| Haiti | 2016 | Folk | 17.0 | 2 |
| Haiti | 2016 | Unaffiliated | 20.2 | 27 |
| Honduras | 2011 | Christian | 0.8 | 14 |
| Honduras | 2011 | Unaffiliated | 1.4 | 4 |
| Honduras | 2011 | Other | 0.0 | 0 |
| India | 2015 | Muslim | 16.0 | 1278 |
| India | 2015 | Christian | 12.1 | 935 |
| India | 2015 | Hindu | 9.3 | 3316 |
| India | 2015 | Unaffiliated | 43.1 | 6 |
| India | 2015 | Other | 3.5 | 85 |
| India | 2015 | Buddhist | 16.3 | 65 |
| Iraq | 2018 | Muslim | 13.3 | 493 |
| Iraq | 2018 | Christian | 14.1 | 2 |
| Iraq | 2018 | Folk | 0.0 | 0 |
| Jamaica | 2011 | Christian | 3.5 | 9 |
| Jamaica | 2011 | Folk | 0.0 | 0 |
| Jamaica | 2011 | Unaffiliated | 4.1 | 3 |
| Jamaica | 2011 | Other | 92.9 | 1 |
| Kazakhstan | 2010 | Muslim | 0.9 | 6 |
| Kazakhstan | 2010 | Christian | 2.6 | 5 |
| Kazakhstan | 2010 | Unaffiliated | 0.0 | 0 |
| Kenya | 2014 | Muslim | 5.6 | 52 |
| Kenya | 2014 | Christian | 2.2 | 71 |
| Kenya | 2014 | Unaffiliated | 2.3 | 5 |
| Kenya | 2014 | Other | 3.5 | 1 |
| Kiribati | 2018 | Christian | 40.6 | 165 |
| Kiribati | 2018 | Unaffiliated | 0.0 | 0 |
| Kiribati | 2018 | Other | 34.4 | 9 |
| Kosovo | 2013 | Muslim | 2.7 | 9 |
| Kosovo | 2013 | Christian | 0.0 | 0 |
| Kyrgyzstan | 2014 | Muslim | 1.6 | 14 |
| Kyrgyzstan | 2014 | Christian | 10.8 | 2 |
| Kyrgyzstan | 2014 | Unaffiliated | 33.2 | 1 |
| Kyrgyzstan | 2014 | Other | 0.0 | 0 |
| Lao | 2017 | Christian | 33.2 | 20 |
| Lao | 2017 | Folk | 36.1 | 385 |
| Lao | 2017 | Unaffiliated | 0.0 | 0 |
| Lao | 2017 | Other | 66.2 | 8 |
| Lao | 2017 | Buddhist | 20.5 | 208 |
| Liberia | 2019 | Muslim | 4.9 | 6 |
| Liberia | 2019 | Christian | 8.7 | 81 |
| Liberia | 2019 | Folk | 17.5 | 2 |
| Liberia | 2019 | Unaffiliated | 23.3 | 3 |
| Madagascar | 2018 | Muslim | 29.1 | 6 |
| Madagascar | 2018 | Christian | 17.7 | 286 |
| Madagascar | 2018 | Folk | 45.5 | 76 |
| Madagascar | 2018 | Unaffiliated | 50.6 | 388 |
| Madagascar | 2018 | Other | 23.4 | 43 |
| Malawi | 2015 | Muslim | 2.8 | 13 |
| Malawi | 2015 | Christian | 2.6 | 62 |
| Malawi | 2015 | Unaffiliated | 0.0 | 0 |
| Malawi | 2015 | Other | 4.6 | 1 |
| Mali | 2018 | Muslim | 17.9 | 407 |
| Mali | 2018 | Christian | 12.3 | 4 |
| Mali | 2018 | Folk | 21.4 | 2 |
| Mali | 2018 | Unaffiliated | 21.7 | 11 |
| Mongolia | 2018 | Muslim | 12.2 | 14 |
| Mongolia | 2018 | Christian | 0.0 | 0 |
| Mongolia | 2018 | Folk | 11.8 | 1 |
| Mongolia | 2018 | Unaffiliated | 1.6 | 13 |
| Mongolia | 2018 | Other | 0.0 | 0 |
| Mongolia | 2018 | Buddhist | 2.8 | 16 |
| Montenegro | 2013 | Muslim | 5.9 | 3 |
| Montenegro | 2013 | Christian | 4.2 | 8 |
| Montenegro | 2013 | Unaffiliated | 0.0 | 0 |
| Montenegro | 2013 | Other | 34.7 | 2 |
| Mozambique | 2015 | Muslim | 4.3 | 7 |
| Mozambique | 2015 | Christian | 10.2 | 50 |
| Mozambique | 2015 | Folk | 12.1 | 16 |
| Mozambique | 2015 | Unaffiliated | 9.5 | 8 |
| Mozambique | 2015 | Other | 42.3 | 6 |
| Namibia | 2013 | Christian | 6.3 | 46 |
| Namibia | 2013 | Unaffiliated | 28.1 | 4 |
| Namibia | 2013 | Other | 13.2 | 7 |
| Nepal | 2019 | Muslim | 23.3 | 15 |
| Nepal | 2019 | Christian | 6.3 | 2 |
| Nepal | 2019 | Hindu | 10.3 | 108 |
| Nepal | 2019 | Folk | 7.2 | 2 |
| Nepal | 2019 | Other | 0.0 | 0 |
| Nepal | 2019 | Buddhist | 5.1 | 4 |
| Nigeria | 2018 | Muslim | 48.5 | 1753 |
| Nigeria | 2018 | Christian | 12.4 | 341 |
| Nigeria | 2018 | Folk | 58.6 | 9 |
| Nigeria | 2018 | Other | 28.8 | 14 |
| North Macedonia | 2018 | Muslim | 7.3 | 4 |
| North Macedonia | 2018 | Christian | 1.5 | 4 |
| Papua New Guinea | 2016 | Christian | 35.8 | 553 |
| Papua New Guinea | 2016 | Unaffiliated | 68.0 | 6 |
| Papua New Guinea | 2016 | Other | 59.1 | 5 |
| Paraguay | 2016 | Christian | 5.1 | 52 |
| Paraguay | 2016 | Unaffiliated | 10.5 | 5 |
| Paraguay | 2016 | Other | 3.1 | 2 |
| Philippines | 2017 | Muslim | 48.1 | 113 |
| Philippines | 2017 | Christian | 11.0 | 218 |
| Philippines | 2017 | Unaffiliated | 37.5 | 3 |
| Philippines | 2017 | Other | 23.5 | 8 |
| Rwanda | 2014 | Muslim | 5.0 | 2 |
| Rwanda | 2014 | Christian | 0.8 | 10 |
| Rwanda | 2014 | Unaffiliated | 10.4 | 1 |
| Rwanda | 2014 | Other | 0.0 | 0 |
| São Tomé and Príncipe | 2019 | Christian | 1.8 | 5 |
| São Tomé and Príncipe | 2019 | Unaffiliated | 6.3 | 3 |
| São Tomé and Príncipe | 2019 | Other | 0.0 | 0 |
| Senegal | 2019 | Muslim | 3.9 | 45 |
| Senegal | 2019 | Christian | 1.6 | 1 |
| Serbia | 2019 | Muslim | 0.0 | 0 |
| Serbia | 2019 | Christian | 3.8 | 15 |
| Serbia | 2019 | Unaffiliated | 0.0 | 0 |
| Serbia | 2019 | Other | 0.0 | 0 |
| Sierra Leone | 2019 | Muslim | 6.1 | 86 |
| Sierra Leone | 2019 | Christian | 2.5 | 10 |
| Sierra Leone | 2019 | Unaffiliated | 0.0 | 0 |
| Suriname | 2018 | Muslim | 2.5 | 6 |
| Suriname | 2018 | Christian | 21.1 | 92 |
| Suriname | 2018 | Hindu | 25.9 | 21 |
| Suriname | 2018 | Folk | 29.5 | 6 |
| Suriname | 2018 | Unaffiliated | 26.6 | 17 |
| Suriname | 2018 | Other | 2.5 | 1 |
| Thailand | 2019 | Muslim | 7.1 | 22 |
| Thailand | 2019 | Christian | 5.5 | 2 |
| Thailand | 2019 | Unaffiliated | 0.0 | 0 |
| Thailand | 2019 | Buddhist | 2.8 | 56 |
| Timor-Leste | 2016 | Muslim | 0.0 | 0 |
| Timor-Leste | 2016 | Christian | 21.6 | 316 |
| Togo | 2017 | Muslim | 12.1 | 24 |
| Togo | 2017 | Christian | 6.6 | 21 |
| Togo | 2017 | Folk | 12.4 | 26 |
| Togo | 2017 | Unaffiliated | 8.6 | 7 |
| Togo | 2017 | Other | 3.4 | 1 |
| Tonga | 2019 | Christian | 3.2 | 6 |
| Tonga | 2019 | Unaffiliated | 100.0 | 1 |
| Tonga | 2019 | Other | 11.1 | 1 |
| Uganda | 2016 | Muslim | 9.3 | 33 |
| Uganda | 2016 | Christian | 4.3 | 108 |
| Uganda | 2016 | Folk | 0.0 | 0 |
| Uganda | 2016 | Unaffiliated | 0.0 | 0 |
| Uganda | 2016 | Other | 4.0 | 1 |
| Vietnam | 2013 | Muslim | 0.0 | 0 |
| Vietnam | 2013 | Christian | 9.1 | 11 |
| Vietnam | 2013 | Unaffiliated | 3.9 | 25 |
| Vietnam | 2013 | Other | 0.0 | 0 |
| Vietnam | 2013 | Buddhist | 0.8 | 1 |
| Zambia | 2018 | Muslim | 0.0 | 0 |
| Zambia | 2018 | Christian | 2.1 | 42 |
| Zambia | 2018 | Other | 0.0 | 0 |
| Zimbabwe | 2019 | Muslim | 0.0 | 0 |
| Zimbabwe | 2019 | Christian | 7.0 | 47 |
| Zimbabwe | 2019 | Folk | 2.8 | 4 |
| Zimbabwe | 2019 | Unaffiliated | 1.2 | 2 |
| Zimbabwe | 2019 | Other | 0.0 | 0 |

Supplementary Table 4. Number of countries with significant differences for each pair of religions with at least 25 children each

| Significant statistical difference in no-DPT prevalence (p < 5%) | Number of countries |
| --- | --- |
| Christian < Muslim | 11 |
| Christian > Muslim | 3 |
| Christian < Hindu | 0 |
| Christian > Hindu | 0 |
| Christian < Buddhist | 0 |
| Christian > Buddhist | 1 |
| Christian < Folk | 3 |
| Christian > Folk | 1 |
| Christian < Other | 0 |
| Christian > Other | 1 |
| Christian < Unaffiliated | 5 |
| Christian > Unaffiliated | 3 |
| Muslim < Hindu | 1 |
| Muslim > Hindu | 2 |
| Muslim < Buddhist | 0 |
| Muslim > Buddhist | 3 |
| Muslim < Folk | 1 |
| Muslim > Folk | 2 |
| Muslim < Other | 1 |
| Muslim > Other | 3 |
| Muslim < Unaffiliated | 2 |
| Muslim > Unaffiliated | 6 |
| Hindu < Buddhist | 0 |
| Hindu > Buddhist | 0 |
| Hindu < Folk | 0 |
| Hindu > Folk | 0 |
| Hindu < Other | 0 |
| Hindu > Other | 1 |
| Hindu < Unaffiliated | 0 |
| Hindu > Unaffiliated | 0 |
| Buddhist < Folk | 1 |
| Buddhist > Folk | 0 |
| Buddhist < Other | 1 |
| Buddhist > Other | 1 |
| Buddhist < Unaffiliated | 1 |
| Buddhist > Unaffiliated | 0 |
| Folk < Other | 0 |
| Folk > Other | 1 |
| Folk < Unaffiliated | 0 |
| Folk > Unaffiliated | 2 |
| Other < Unaffiliated | 3 |
| Other > Unaffiliated | 0 |

Supplementary Table 5. Categories of the original variables recoded into eight religious groups, Demographic and Health Surveys and Multiple Indicator Cluster Surveys

| Religious group | Original categories | | | | | | | | |
| --- | --- | --- | --- | --- | --- | --- | --- | --- | --- |
| Christian | adventist | adventist/jehova | Adventista | Adventiste | Aglipay | Anglican | anglican church | apostolic sect | Armée de salut |
|  | Armee du salut | Assemblée de Dieu | assembly of god | Autre chrétien | Autre religion chrétienne | Autre religion chrétienne (préciser) | Autres chrétiens | Autres chretiens independants | Autres protestants |
|  | baptist | Catholic | Catholique | Católica | Catolica | Católica | ccap | Céleste | Celestes (Celestial Church of Christ) |
|  | chistiane | Chrétien | Chrétienne | Christian | Christian Catholic | Christian Protestant | Christianity | Cristiana | Deeper Life |
|  | eglise de réveil | Eglises de reveil | Eglises de réveil | elcin | evangelic | Evangélica | Evangelical | Evangelical / Protestant | evangelical presbyterian |
|  | Evangelical/pentecostal | Evangélico | Evangélique | Evangelique Presbytérienne | Evangélica | Harriste | Hristian | iglesia ni cristo | Iglesia Ni Kristo |
|  | jehovah witness | jehovah's witness | jehovah's witnesses | lesotho evangelical church | Mennonite | Methodist | Méthodiste | Mormón | Mormón |
|  | Nazarene | Nova apostólica | orthodox | Orthodox Christian | Ortodox | Ortodox Christian | Other Christian | Other Christian (specify) | Other christians |
|  | other chritians | Other Protestant | other religions | pentecostal | pentecostal/born again/evangelical | pentecostal/charismatic | pentecotist | presbyterian | Protestant |
|  | Protestant méthodiste | protestant/ other christian | protestant/anglican | Protestante | Roman Catholic | roman catholic church | Salutiste | salvation army | sda |
|  | seventh day advent/ baptist | seventh day adventist | seventh day adventist / baptist | seventh-day adventist | Temoins de Jehovah | Testigo de Jehová | Testigo de Jeová | universal | Free wesleyan church |
|  | united church | Free church of tonga | Latter day saints | Tokaikolo/maamafo’ou |  |  |  |  |  |
| Muslim | Islam | Islamic | Muslim | Moslem | muslin | Musulman | Musulmane | Muçulmano | Musulmanne |
| Hindu | hindu | hinduism |  |  |  |  |  |  |  |
| Buddhist | buddhism | buddhist | buddhist/neo-buddhist | Budda | Buddism | Hoa Hao |  |  |  |
| Folk/ Traditional | Animist | animiste | indigenous spirituality | kibanguist | Kimbanguiste | kirat | Other traditional | parsi/zoroastrian | sect |
|  | tradition/animist | Traditional | Traditional religion | traditional/animist | traditional/spiritualist | Traditionalist | Traditionnal/animist | vaudousant | Vodoun |
|  | zephirrin/matsouanist/ngunza | Zion | Anemista | Animisme/ Religions traditionnelles afric. | Autres traditionnelles |  | Kimbaguiste | Rastafarian | Rastafarianism |
|  | Shamanism | Shamanist | Traditionnelle / Animiste | Azidi/Yazidism |  |  |  |  |  |
| Other | Bahai | baha'i | Bundu dia kongo | jain | mammon | Other | Other religion | sikh | Vuvamu |
|  | Autre | Autre religion | Autre religion (non chrétien) | Autre religion (préciser) | Bon | Cao Dai | Other religion (specify) | Others | Otra religion |
|  | Otra religión | Otras religiones | Outra religião | Prakriti | Spiritualist | Non-christian | SABE’E/Sabianism |  |  |
| Unaffiliated | No religion | None | Does not hold any religion | Ninguna religión | no religión | Pas de religion | Sans religion | Sem religião |  |
